# Simulator-Based Metrics for Quantifying Vascular Palpation Skill for Cannulation

**DOI:** 10.1109/access.2022.3184303

**Published:** 2022-06-20

**Authors:** LYDIA PETERSEN, ZHANHE LIU, JOE BIBLE, DEVANSH SHUKLA, RAVIKIRAN SINGAPOGU

**Affiliations:** 1 Department of Bioengineering, Clemson University, Clemson, SC 29634, USA; 2 School of Mathematical and Statistical Sciences, Clemson University, Clemson, SC 29634, USA

**Keywords:** Medical education, palpation, simulation, skill assessment

## Abstract

Palpation is essential for accurate diagnosis and treatment in many clinical examinations and procedures. Specifically, vascular palpation is used to diagnose cardiovascular health issues and identify anatomical landmarks in the peripheral vascular system. However, little attention has been given to quantifying what comprises skilled vascular palpation; therefore, this study aims to objectively quantify the differences between high performer (HP), mid performer (MP), and low performer (LP) behavior towards understanding vascular palpation skills. Eleven HPs, twenty-five MPs, and ten LPs completed sixteen trials on our simulator under various conditions. There were four fistulas, two skin thicknesses, and two motor vibration intensities. Finger force and location data were recorded for each trial on the simulator. We examined three types of palpation metrics: time, force, and location. All three types of metrics demonstrated statistically significant differences between HP and LP palpation behavior. Therefore, these metrics could be used for structured and standardized palpation skills training in the future, potentially improving patient outcomes.

## I. INTRODUCTION

Palpation is the process of using a hand for touching or feeling to find abnormalities or to identify physiological landmarks below the skin surface. Clinical palpation is used in many medical disciplines, from athletics training [1] and veterinarian practices [2] to medical examinations [3]–[5] for medical diagnosis and to assess if certain pathologies are present. There are two main kinds of palpation: stiffness-based and vascular. Stiffness-based palpation is often used to identify pathological tissues’ size, shape, stiffness, and location [6]. The relationship between a person’s exploratory techniques and their efficacy is a matter of current study, for example, different methods of palpation movement [7]. An example of stiffness-based palpation is its use in breast palpation for identifying tumors [5], [8]–[10].

The other type of palpation–Vascular palpation–is also widely used in medical examinations. In typical vascular palpation, one or more fingers are used to feel for haptic stimulus from blood vessels that includes perceiving the quality of blood flow from vibratory stimulus as well as the geometrical structure of vasculature. Discerning the quality of blood flow based on touch feedback makes vascular palpation unique. One example of this type of palpation is the Peripheral Vascular (PVS) Examination [11], examining patients’ risk for peripheral vascular disease. Another medical procedure where vascular palpation is critical is cannulating for hemodialysis. This procedure is critical because patient survival depends on successful cannulation of their vascular accesses thrice a week. This procedure is notably challenging because the blood vessel typically cannulated (an arteriovenous fistula, AVF) is a patient-specific anatomical structure. An AVF is created by surgically connecting a vein to an artery, which can mature into many shapes and sizes. Furthermore, once connected, the blood flow in the AVF evolves, often resulting in a turbulent and high volume of blood flow through the fistula. Consequently, learning to palpate AVFs accurately for successful hemodialysis is a complex and critical skill that needs targeted training [12], [13].

Simulators have been widely applied for medical skills training and assessment, particularly as simulators allow for objective skill quantification, which is essential for skill assessment and training [14]. Objective metrics are valuable for identifying skill improvement because they enable tracking trainee progress over time [16]–[19]. Additionally, simulator-based training allows for structured skills training in a low-stakes, non-clinical environment, reducing training times and patient risks. There is also the potential to use simulators to tailor an individual’s training to target specific weaknesses. While simulator-based training has been successfully applied in surgical disciplines [20]–[22], it has not received much attention for teaching palpation.

In general, there are three types of sensor-based palpation simulators that measure performance: virtual, hybrid, and physical. Virtual, and sometimes hybrid, simulators often use one or more haptic devices to obtain physical input and render haptic feedback (with the visual feedback often displayed on a monitor) [23]–[25]. Virtual simulators benefit from the ability to create multiple simulation scenarios by adjusting various hardware and software settings with a click of a button. Nevertheless, they can malfunction during practice [26] and are limited in realism since users cannot have a fully multimodal experience as with physical simulators. Physical simulators, on the other hand, often feature benchtop models (e.g., synthetic organs or surgical materials) with standard medical tools used in procedures. These models can incorporate sensors to record specific measurements pertinent to skilled action to differentiate high and low performer skill [5], [27]. In other cases, users were outfitted with sensors as they performed tasks on a simulator [30]. These simulators typically incorporate force or pressure sensors; for instance, Laufer and colleagues used an array of piezoelectric sensors under the palpation surface [31] while Granados and colleagues outfitted the users’ fingers with sensors during palpation. Some of the earliest work in palpation skill assessment through sensor recordings was performed on a benchtop model studying pelvic exams [27], where the researchers used force sensors located at strategic points to quantify palpation. Later, clinical breast examination (CBE) and digital rectal examination (DRE) simulators were created using this same approach [5], [8], [32]. These studies have demonstrated that simulators can be effective in assessing aspects of palpation skill. However, all of these studies have been concerned with stiffness-based palpation.

In contrast, little attention has been given to systematically and quantitatively studying vascular palpation. A few recent studies explored custom-designed simulator methods for vascular palpation, emphasizing hardware and software development. John and colleagues developed a simulator for femoral artery palpation for arterial catheterization, utilizing a mixed-reality setup [33]. This group attempted to retain the realism of a tangible membrane for needle insertion while also creating flexibility by using haptic feedback to create an augmented reality. However, the goal was to show the feasibility of using such a simulator and not to measure its ability in skills assessment or training. Hung *et al*. presented an ultrasound-based method for rendering pulsatile feedback that could be used in vascular simulation applications [34]. These two studies address potential technologies that could be used to render feedback useful for vascular palpation simulators. However, to our knowledge, no study has examined the quantitative differences between high and low skill for vascular palpation. Consequently, there is a need for a systematic examination of vascular palpation skill.

Current research does not have a clear definition or quantitative measures for skilled performance in vascular palpation. The following are salient features of vascular palpation that must be identified and measured in a suitable simulator. The haptics arising from blood flow must be rendered in a human-discernible way. That is, pulsatile or turbulent blood flow must be haptically rendered in the simulator. In addition, vascular palpation also includes searching for specific blood vessels that are often inconspicuous and not easily identifiable. In this study, we examine palpation for hemodialysis cannulation–a procedure where vascular palpation plays a key role. As previously mentioned, cannulation to initiate dialysis is particularly difficult because vascular accesses (typically AVFs) are in irregular geometries with varying blood flows. There is a pressing clinical need to avoid miscannulation in hemodialysis since it can lead to excessive bleeding, the destruction of the vascular access, and thrombosis [35]. As such, there is a need to train clinicians to palpate vascular accesses safely and effectively.

Building on our previous work [36], this study aims to (1) identify metrics which objectively differentiate between high performer (HP), mid performer (MP), and low performer (LP) behavior during cannulation towards quantitatively understanding vascular palpation skill and (2) to relate these metrics to objective cannulation outcome measures that were previously described [37], [38]. To our knowledge, for the first time both force and motion sensor data are used to understand vascular palpation skill specifically.

## II. METHODS AND PROCEDURES

### A. SIMULATOR

**FIGURE 1.**
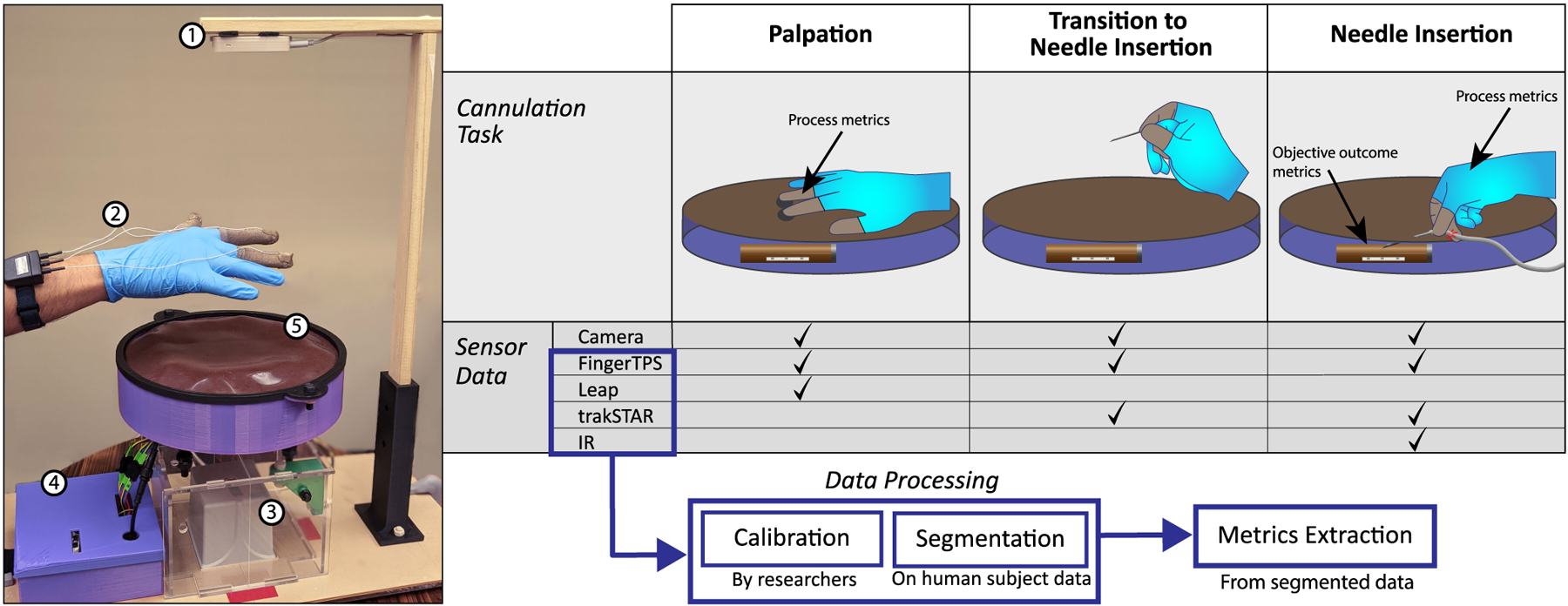
(1) Shows the setup of the hemodialysis cannulation simulator: [1] Leap Motion Controller, [2] FingerTPS, [3] trakSTAR [4] Control Box, [5] Simulator Bed. (2) The cannulation task divided into phases (row 1), the sensor streams of primary importance for each phase (row 2), and an overview of data processing (bottom).

This study examines subject data collected from a novel hemodialysis (HD) cannulation simulator [37], which has been previously demonstrated for successful quantification of cannulation skill [38]. The simulator itself is comprised of four fistulas located radially around the simulator bed with motors in each fistula vibrating at an intensity and pattern to simulate “thrill” at the point of anastomosis, where the fistula was created. The vibration pattern was generated by converting an audio recording of an anastomosis into vibration intensity. The system contains five sets of sensors: a Leap Motion Controller for tracking the hand (Ultraleap Inc.), FingerTPS to record palpation forces (Pressure Profile Systems Inc.), trakSTAR electromagnetic (EM) position sensor for tracking needle position (Northern Digital Inc.), and infrared (IR) emitters and detectors for determining if the needle is inside the fistula. The Leap Motion Controller is fixed above the simulator to a frame, the FingerTPS sensors are fitted onto participants’ thumb, index, and middle fingers, and the IR sensors are embedded within the needle tip and the fistulas in the simulator. The EM sensor’s field generator is positioned under the simulator, with its corresponding sensor embedded in the needle tip alongside the IR sensor. Finally, an external Intel RealSense camera records video of the subject performing the task. The skin and fistula models in the simulator were created using cured silicone (Ecoflex; Smooth-On Inc.). Fig. 1 (1) shows the simulator with its features labeled.

### B. EXPERIMENTAL PROTOCOL AND DATA COLLECTION

Ethics approval for this study was provided by the Institutional Review Boards (IRB) of Clemson University and Prisma Health (IRB number: Pro00064701). Participants for this study were recruited at a regional meeting of dialysis clinicians. No subject had previous experience cannulating using the device. Nurses and technicians at this conference first went through a PowerPoint presentation with instructions on using the simulator and filled out a demographics questionnaire. Each subject performed 16 trials on the simulator to allow for different scenarios. There were four fistulas, two different skin thicknesses, and two different motor vibration intensities, and the order of fistulas and their intensities were randomized to provide a unique experience for each trial in an attempt to minimize learning effects. Finally, subjects completed a post-experimental questionnaire, scoring the simulator’s realism and usefulness for training. Peer-recognized experts observed participants as they performed the trials and rated each subject on a global rating scale (GRS) on a scale of 1–7 in five categories [38]. Only two categories could be argued to be relevant to palpation (palpation skill and overall), while the other categories focus on needle insertion. Those who received a score of 7 in palpation and overall skill were categorized as HPs for this analysis. Those who received a score of 5 or less in palpation skill and 4 or less in overall skill were categorized as LPs. Among a total of 49 cannulators, 11 HPs and 10 LPs fit these criteria with a mean of 11.2 and 11.5 years of cannulation experience for HPs and LPs, respectively. Participants who did not fall into these categories were labeled as MPs, who had an average of 11.8 years of cannulation experience.

Each trial consists of two major parts (see Fig. 1 (2)). The first is palpation, where participants were asked to identify the location and orientation of the fistula of interest. Then, participants were instructed to insert the needle to obtain blood flashback, as indicated by a red LED above the needle. This paper examines palpation, the first part of each cannulation trial. Consequently, segmentation was performed to isolate sensor data during the palpation portion of the trial.

After segmentation, a dataset containing 171 trials from HPs, 363 from MPs, and 153 trials from LPs was identified according to the criteria defined in the next sections. In addition, a subset of 70, 117, and 41 trials were examined to compare location-based metrics for HPs, MPs, and LPs, respectively. Details about segmentation procedures and criteria are described in subsection C-2.

### C. DATA PROCESSING

#### 1) CALIBRATION

We performed calibration to determine the locations of the four fistulas in the simulator to extract location-specific metrics. Towards this objective, the following calibration procedure was performed before collecting experimental data. First, one experimenter traced an index finger along the central axis of each fistula while recording Leap data. Calibration was performed without the skin layer, leaving the four fistulas exposed and surrounded by foam. Following this, these data from the Leap sensor were fitted to lines that approximated the respective fistula’s central axes. We observed that the quality of calibration data depended on various factors, such as the material reflective properties pertinent to NIR-based camera detection during the calibration process.

**FIGURE 2.**
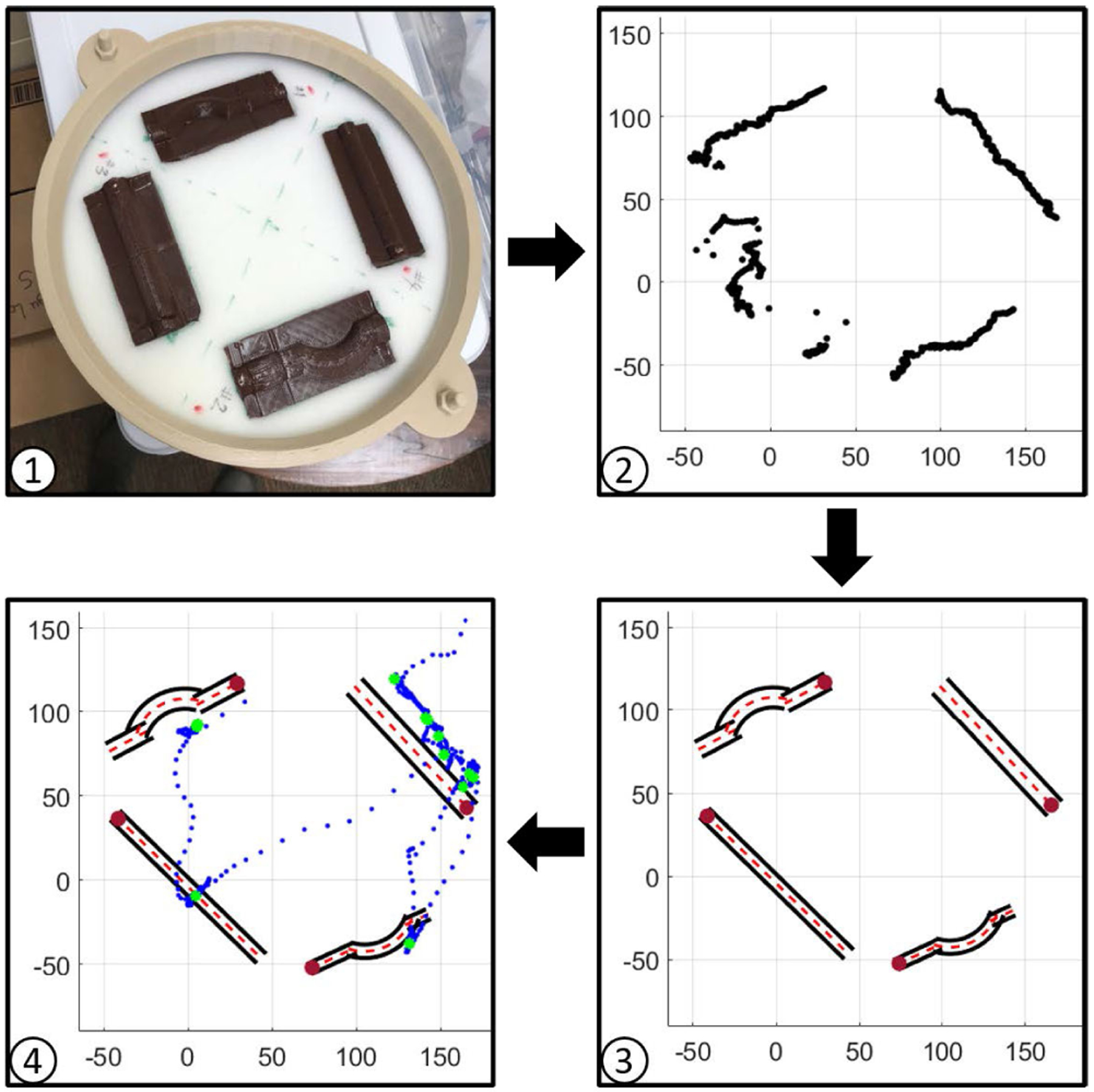
Calibration process (1) the simulator bed (2) raw data collected from the Leap sensor (3) estimated geometry of fistulas (4) example of user’s location data (blue) and touchpoints (green) with respect to the fistulas.

In this study, we used the location of anastomosis (rendered by a vibration motor) to compute the four location-based metrics defined in the next section. Also, as shown in Fig. 2 (4), palpation trajectories were plotted with respect to fistula locations according to calibration to gain insight into patterns of palpation behavior. The methods presented here could be used for extracting more location-based metrics in the future, such as estimating fistula orientation.

#### 2) SEGMENTATION

**FIGURE 3.**
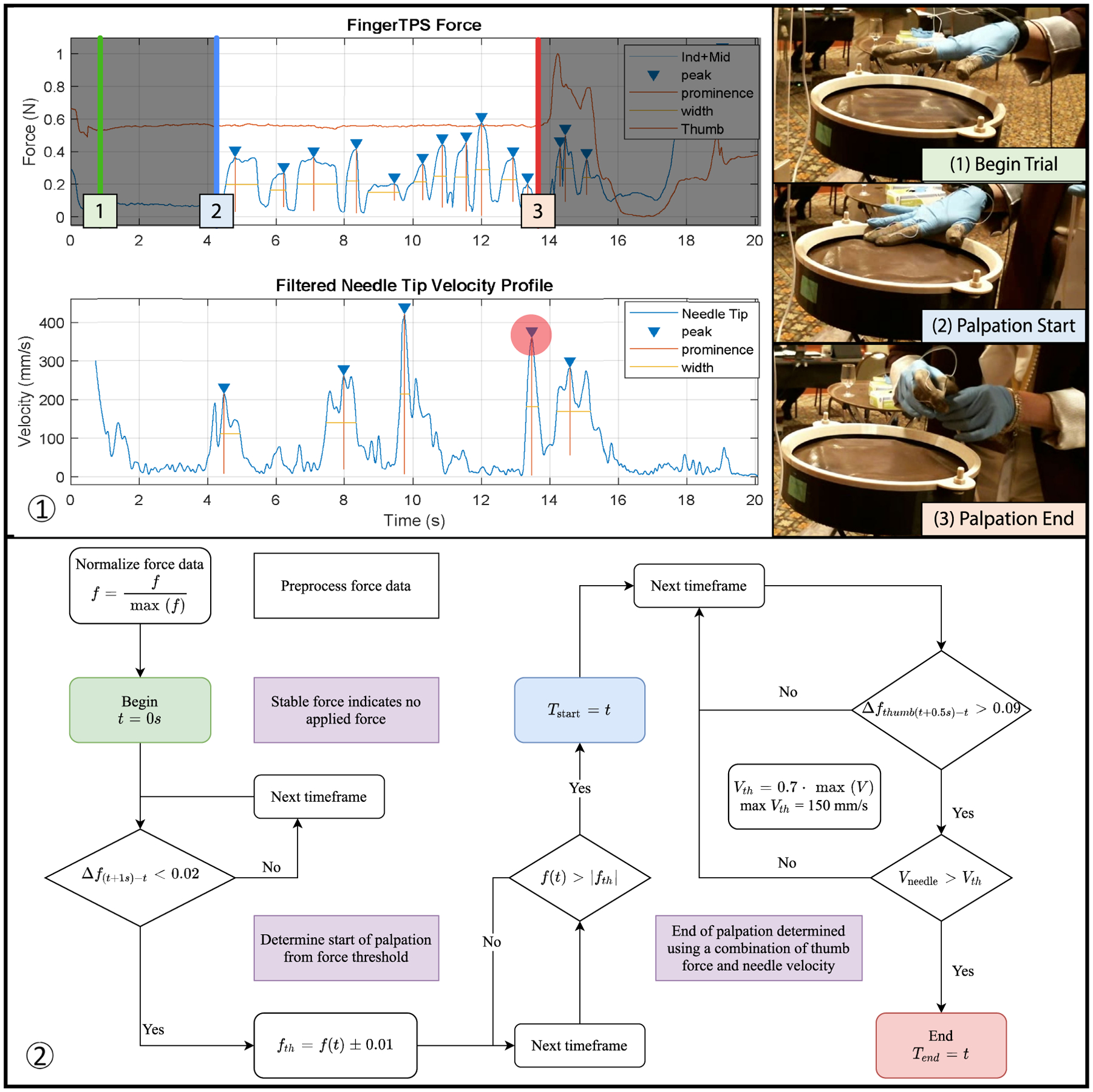
**Segmentation process for isolating palpation from the rest of the task. (1) Example of segmentation using user data and (2) the flowchart of segmentation**.

An overview of the segmentation algorithm can be seen in Fig. 3 (1). The start time of palpation (*T*_*start*_) was defined as the time when force was applied to the index or middle finger after a period of no change in force. The initial period of accounting for no change in force is designed to detect trials where the user was not in the starting hand position. The end time of palpation (*T*_*end*_) was found by searching for a change in force applied by the thumb in conjunction with a movement of the needle. Typically at the end of palpation, the needle will be gripped by the subject for insertion, resulting in pinch forces and needle movement. This segmentation strategy works for the typical palpation trial, as demonstrated in Fig. 3, (1), where the subject palpates with the index and middle fingers while the needle is held or placed relatively still.

However, not all trials were successfully segmented by the algorithm due to various atypical palpation and needle holding behaviors. Most of these errors are due to the subject holding the needle with the hand that is not palpating in preparation for needle insertion. As a result, detecting needle movement in the algorithm prematurely triggers the end of palpation. Other reasons for incorrect segmentation are: subjects palpating with the incorrect hand (without the force sensors), picking up the needle before completing their palpation, or using unusual palpation strategies like palpating nearly exclusively with the thumb. For trials where automatic segmentation was not possible, but the data collected were still valuable for metrics extraction, we manually observed the captured videos to identify the timestamps for the start and end of palpation.

We excluded trials with missing sensor data from this analysis. As a result, a dataset containing 171 trials from HPs, 363 trials from MPs, and 153 trials from LPs was identified to analyze force-based metrics described in the following section. These metrics do not require any location data; as such, trials without stable finger location data could still be included. From these trials, only those with all position data during palpation were included to analyze location-based metrics resulting in a subset of 70, 117, and 41 trials for high, mid, and low performers, respectively.

### D. PALPATION METRICS

Metrics are split up into three types, force metrics, location metrics, and time metrics.

#### 1) FORCE METRICS

Per touchpoint:

- Touchpoint Time (*TPT*) is the dwell time, or the amount of time the subject spends at each touchpoint and is found by the width of the force peak found with MATLAB’s *findpeaks* function.
- Touchpoint Force (*TPF*) is the force applied by the user’s index and middle fingers (ind+mid) at a touchpoint, *F*_*ind*+*mid*_ at *t*_*tp*_, where *t*_*tp*_ is the timestamp of each of the peaks identified by the *findpeaks* function.

Per trial:

- *Touchpoints* is the total number of touchpoints during palpation, defined by the number of peaks in the force profile of the subject and indicates the number of times there was applied pressure to the surface of the simulator bed during palpation.
- Touch Frequency (*Frequency*) is the number of touchpoints recorded per second,
(1) $$\text{Frequency}=\frac{\text{Touchpoints}}{T_{\text{end}}-T_{\text{start}}}$$

- *Total Force* is calculated by summing up an estimate of the forces applied during a touchpoint. The estimate is calculated by multiplying the peak force at the touchpoint (*TPF*) by the Touchpoint Time (*TPT*).
(2) $$\text{Total}\;\text{Force}=\sum_{\text{tp}=1}^{\text{Touchpoints}}\text{TP}T_{\text{tp}}*\text{TP}F_{tp}$$

- *Force Range* is the difference between maximum and minimum forces applied during a trial.
(3) ForceRange=max(Force)−min(Force)

#### 2) LOCATION METRICS

Per touchpoint:

- Distance to motor (*TPD*) is the distance from a touchpoint to the motor that is activated,
(4) $$\text{TPD}=\sqrt{{\left(x_{tp}-x_{m}\right)}^{2}+{\left(y_{tp}-y_{m}\right)}^{2}}$$

Per trial:

- The Ratio of Correct Movement (*RCM*) is defined by the number of velocity projections that are in the direction of the motor over the total number of significant movements:
(5) $$\begin{array}{l} RCM=\frac{\sum_{n=1}^{T}f\left(V_{p}(n)\right)}{\sum_{n=1}^{T}f\left(\left|V_{p}(n)\right|\right)}*100\%\text{ where ,}\\ f\left(V_{p}(n)\right)=\{\begin{array}{ll} 1, & \text{if }V_{p}(n)>20\text{mm/s}\\ 0, & \text{otherwise} \end{array}\;\text{where}, \end{array}$$
  *V*_*p*_(*n*): projected velocity at time frame n, see [36]

- The Ratio of Near Touchpoints (*RNTP*) is the number of touchpoints within 40 mm of the motor (simulating anastomosis) over the total number of touchpoints:
(6) $$\text{RNTP}=\frac{\sum_{\text{tp}=1}^{\text{Touchpoints}}f\left(TPD_{tp}\right)}{\sum_{\text{tp}=1}^{\text{Touchpoints}}TPD_{tp}}*100\text{\% where, }\;f\left(TPD_{tp}\right)=\{\begin{array}{ll} 1, & \text{if }TPD_{tp}<40\text{mm}\\ 0, & \text{otherwise} \end{array}$$

- Path Length is the total distance the index finger moves during palpation:
(7) $$PL=\sum_{n=1}^{T-1}\sqrt{{\left(x_{n+1}-x_{n}\right)}^{2}+{\left(y_{n+1}-y_{n}\right)}^{2}}$$

#### 3) TIME METRICS

The *Dwell Time* is the total time that the subject is touching the skin. It is the sum of *TPT* per trial.


(8) $$\sum_{\text{tp}=1}^{\text{Touchpoints}}TPT_{tp}$$

*Idle Time* is the total time from the start of palpation to the end that is not touching the skin.


(9) $$\text{Idle}\;\text{Time}=\left(T_{\text{e}nd}-T_{\text{start}}\right)-\text{Dwell}\;\text{Time}$$

### E. OUTCOME METRIC

In recent literature, there has been an attempt to correlate process metrics obtained during clinical performance (on simulators or in the clinic) with clinical outcomes [39]–[41]. Our group has developed an objective metric that quantifies the outcome of the cannulation task on our simulator. Since this would more closely affect clinical outcomes and, consequently, is of interest to clinicians.

As described in our earlier work, we used *stb*, the indicator of whether or not stable flashback was achieved upon cannulation as our outcome metric in this study. *stb* = 1 when “there is at least 2 s of flashback without any interruption until the end of a trial” [38]. All other trials had *stb* = 0. This metric was used as an outcome metric since it corresponds to the clinical scenario where successful cannulation means sustained blood flow for hemodialysis.

## III. RESULTS

### A. COMPARING PALPATION METRICS ACROSS SKILL LEVELS

**FIGURE 4.**
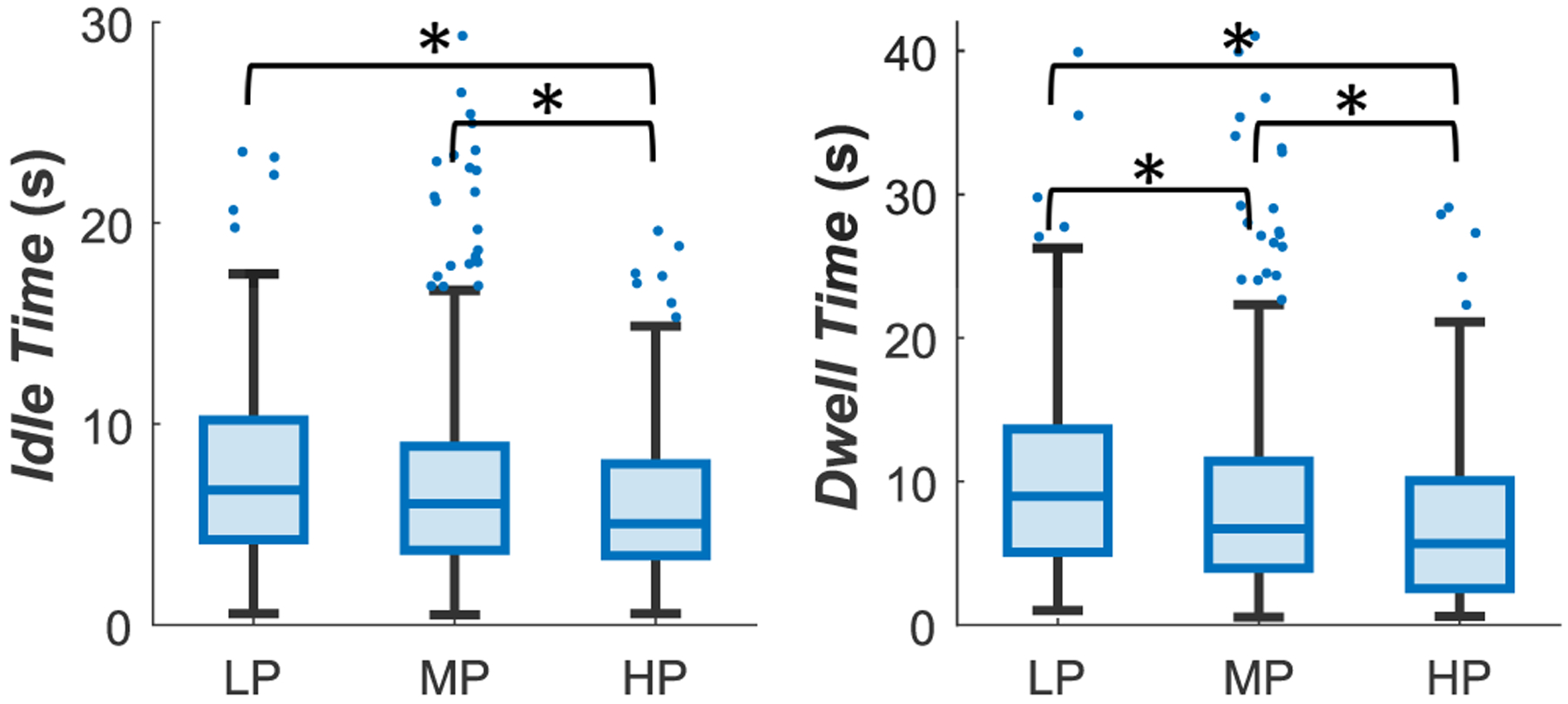
Boxplot of the Time metrics. * shows that the distribution of values in one group is, on average, larger than the other.

A summary of the results from this portion of the study can be seen in Table. 1. *Dwell Time*, the total time spent applying forces during palpation, had statistically significantly lower medians for HPs campared to LPs (~5.7 s vs. 9 s), see Fig. 4. Similarly, the total *Idle Time*, *i.e*., the median time spent not palpating, was statistically significantly lower for HPs than LPs (~5 s vs. 6.7 s). These results suggest that palpation time is an important factor for palpation skill assessment.

**FIGURE 5.**
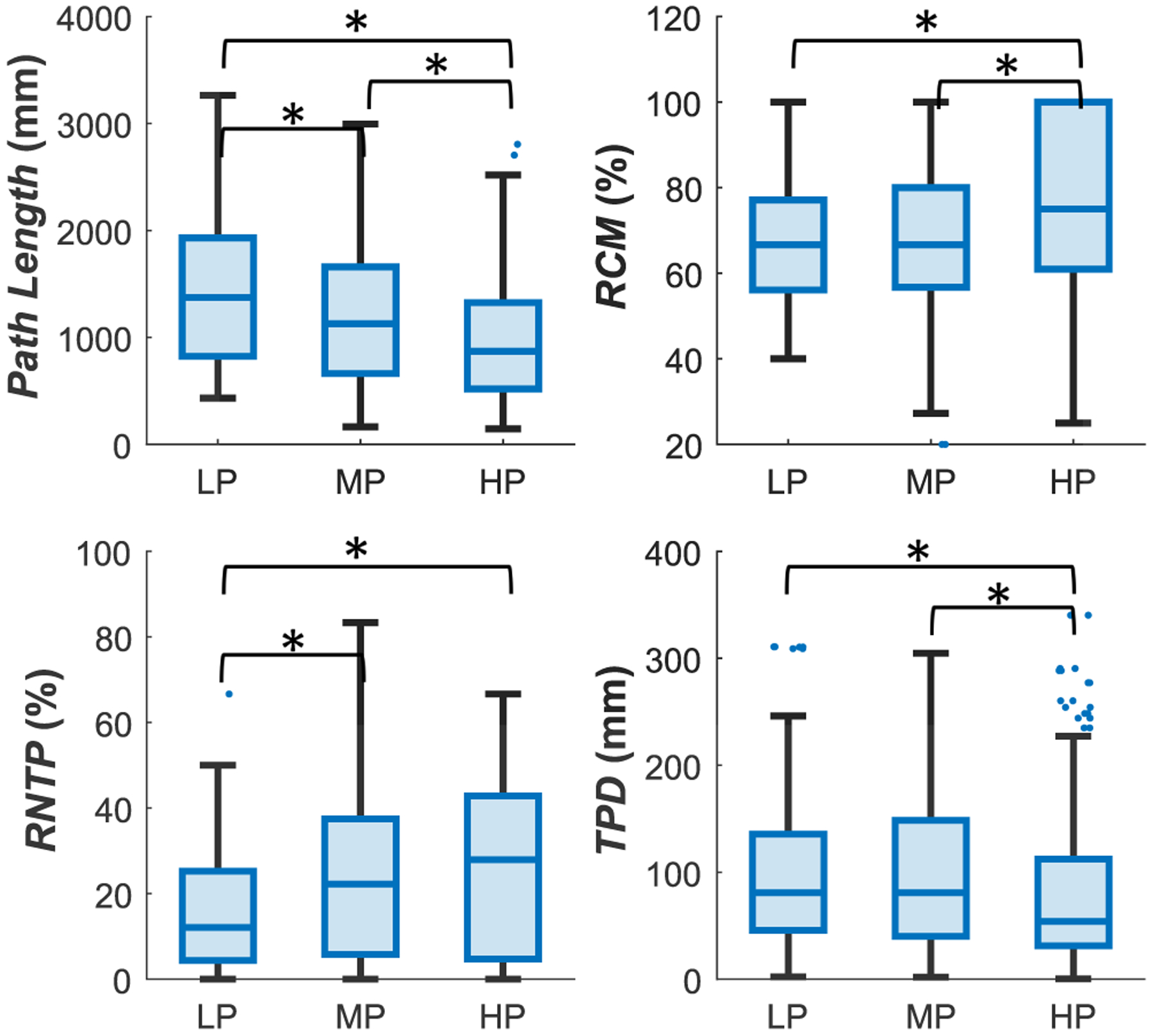
Boxplots of location metrics. No less than 98% of the data are plotted, and the remaining outliers are beyond the axes shown for better visualization. * shows that the distribution of values in one group is, on average, larger than the other.

All location metrics demonstrated statistically significant differences between HPs and LPs, as shown in Fig. 5. HPs had statistically significantly shorter median *Path Length* (the total distance traversed during palpation) than LPs (~0.9 m vs. 1.4 m). HPs also demonstrated efficiency of palpation movement as indicated by the *RCM* and *RNTP* metrics: HPs demonstrated movement toward the point of anastomosis 3/4th of the time (versus 2/3rds for LPs) and HPs had 15.8% more touchpoints than LPs that were close to the anastomosis. In addition, the HPs also palpated closer to the point of anastomosis per each touchpoint, as indicated by the *TPD* metric.

**FIGURE 6.**
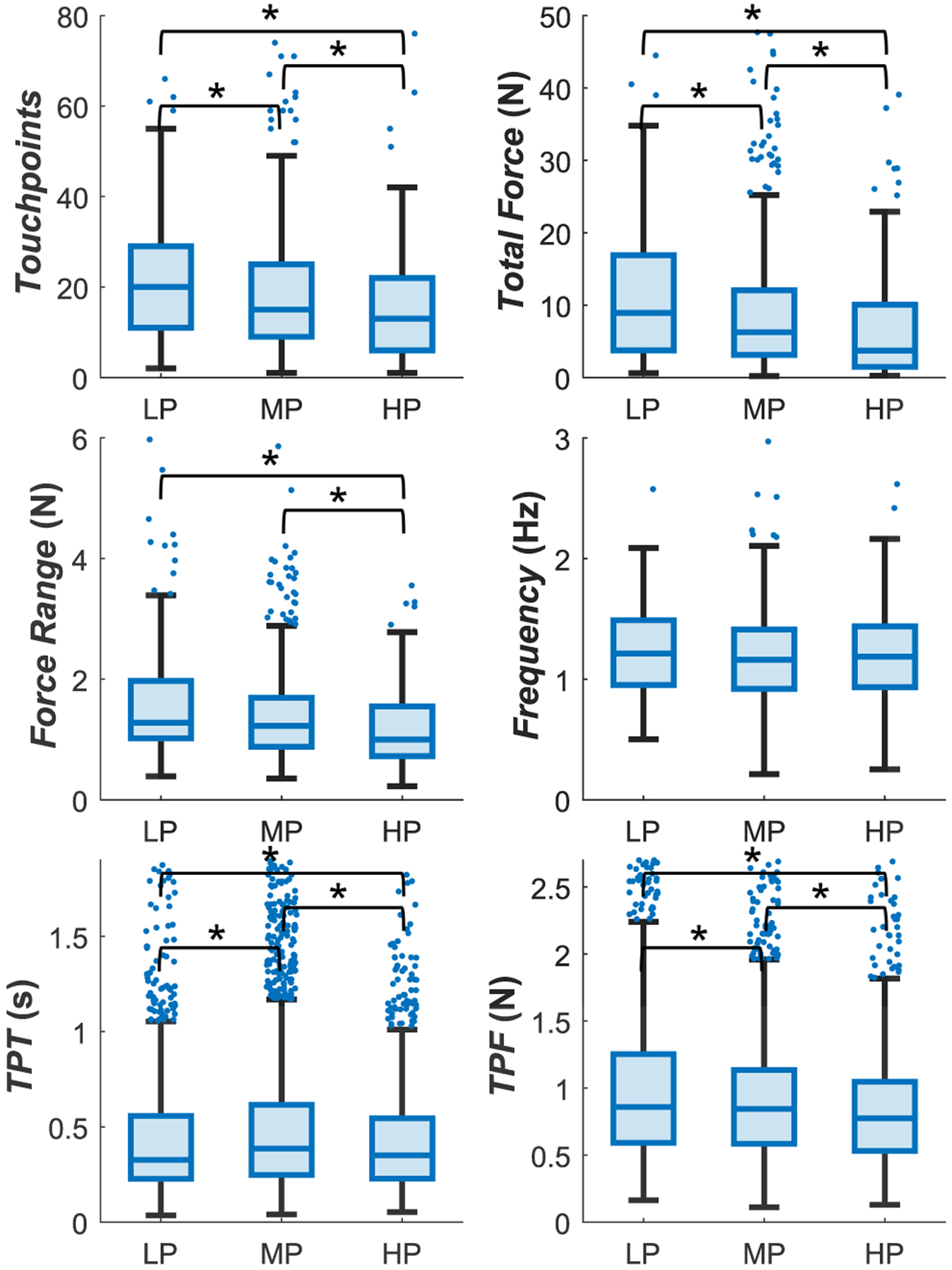
Boxplots of Force metrics. No less than 98% of any the data are plotted, and the remaining outliers are beyond the axes shown for better visualization. * shows that the distribution of values in one group is, on average, larger than the other.

Five out of the 6 force metrics also demonstrated statistical difference between HP and LP behavior, which can be seen in Fig. 6. Not only did HPs have a lower number of *Touchpoints* than LPs, but they also applied gentler forces, as indicated by the *Total Force* and *TPF* metrics. This observation validates anecdotal reports from expert nurse educators suggesting that experts have “light hands” during cannulation. Since cannulation directly influences patient experience, clinicians who apply greater forces might risk providing an unpleasant patient experience. Furthermore, HPs were more consistent in the forces they applied, as shown by their *Force Range*. HPs also dwelt statistically significantly longer at individual touchpoints (*TPT*) than LPs, suggesting greater intentionality.

As indicated in Table 1, we also calculated differences in all metrics for LPs versus MPs as well as MPs versus HPs. As described in our methodology, we used expert ratings to determine HPs and LPs first. Those who did not fit in either group were classified as MPs. Consequently, MPs included a wider spread of skill with regard to palpation behavior. When comparing LPs to MPs, 7 out of the 12 metrics demonstrated statistical significance, while 10 of the 12 metrics demonstrated statistical significance between MPs and HPs.

### B. PALPATION METRICS AND CANNULATION OUTCOMES

We computed the probability of successful cannulation (based on the *stb* outcome metric) for the three groups of subjects: LPs, MPs, and HPs. The mean *p*(*success*|*LP*) = 0.548 (95%CI 0.482, 0.614), indicating that the likelihood of this group having a successful cannulation is similar to that of a coin toss. HPs, in contrast, have a likelihood of success close to 1, *p*(*success*|*HP*) = 0946 (95%CI 0.902, 0.971), which suggests that HPs’ palpation behavior almost certainly leads to successful cannulation. From the univariate models for HPs that predict the probability of success from our suite of force metrics, we observed that no force metric was a statistically significant predictor of *p*(*success*) (see Table. 2). As such, we infer that the variability seen from the HP metrics does not significantly affect their *p*(*success*). The mean *p*(*success*|*MP*) = 0.850 (95%CI 0.811, 0.881).

**FIGURE 8.**
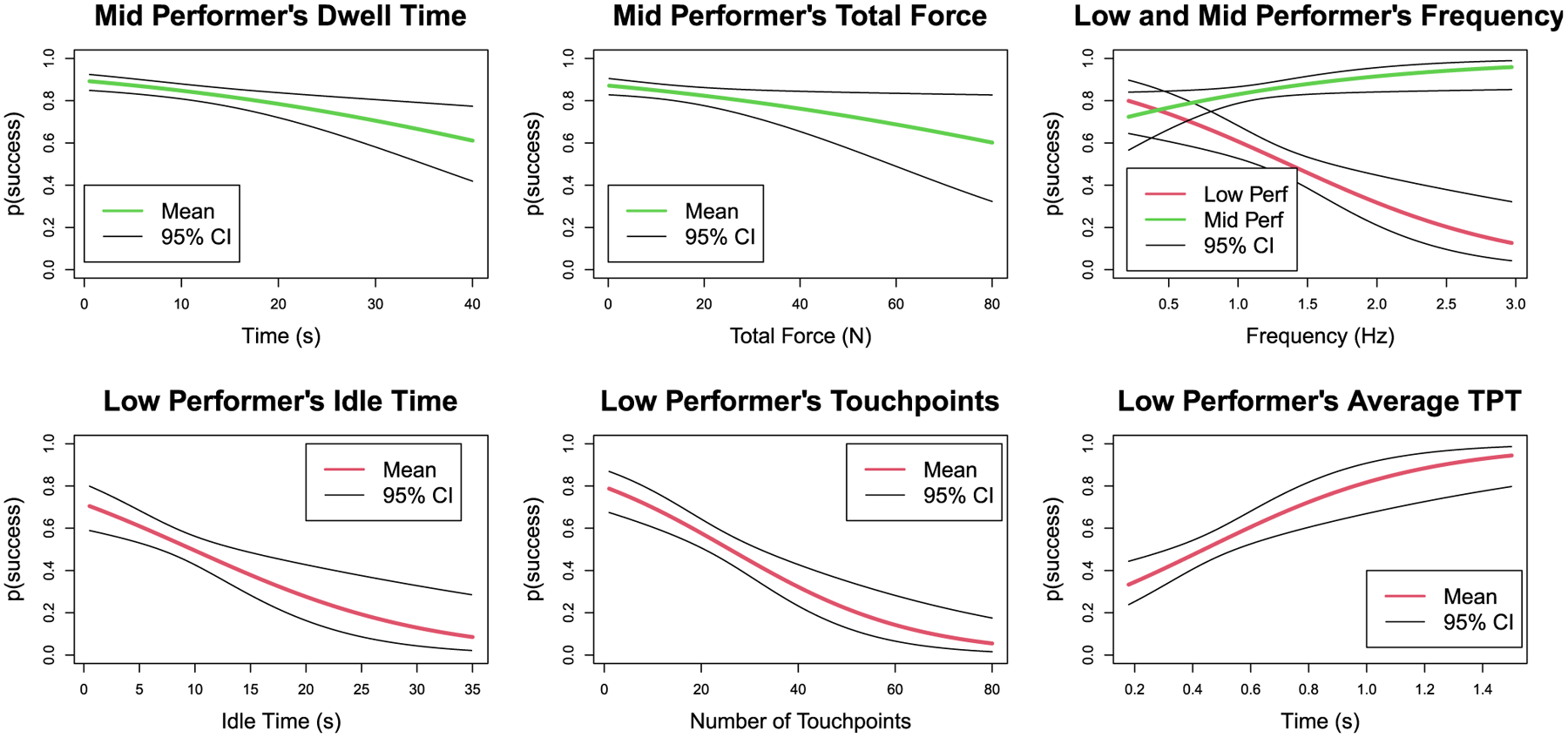
**Hill functions of the statistically significant features from the uni-variate models**.

For the LP and MP groups, certain force metrics predict the probability of successful cannulation. Hill functions relating statistically significant metrics to the *p*(*success*) are given in Fig. 8. If *Idle Time* is greater than 20 s, then *p*(*success*) decreased rapidly. Similarly, as the number of *Touchpoints* increased, *p*(*success*) also decreased. In contrast, as time spent per touchpoint (*TPT*) increased, the probability of successful cannulation was improved. These observations could have important implications for training.

For the MP group, we observed that as *Dwell Time* and *Total Force* applied during a trial increased, the probability of successful cannulation decreased. The *Frequency* metric, which measures the number of touchpoints per second, demonstrated an interesting contrast between LP and MP groups. For LPs, as *Frequency* increased, *p*(*success*) decreased, whereas, for MPs, *p*(*success*) remained relatively constant after a frequency of 1.5 Hz. This observation could suggest that palpation at higher frequencies among LPs could indicate their uncertainty during the palpation process. On the other hand, MPs seem more certain despite their variation of frequency of palpation.

## IV. DISCUSSION

In vascular palpation, blood-flow-induced vibration is used as a guide to locate areas of interest in the vascular system and assess its health. Simultaneously, touch stimuli are used to determine geometric properties of blood vessels (e.g., diameter, depth). Vascular palpation is often a means to an end, wherein the goal of the procedure requiring palpation is to insert a needle or cannula into a patient’s vascular access. In this study, our cannulation simulator was custom-built to study both the palpation and needle insertion aspects of cannulation, specific to hemodialysis. For this procedure, the first step is assessing the health of a patient’s vascular access, typically an AVF, using palpation. To better study palpation and needle insertion skills, our simulator was designed to have a flat, circular surface rather than mimicking the anatomical structure of an arm, which would provide geometrical clues on vessel location. As noted earlier, the simulator featured four fistulas with varying characteristics, so users must rely predominantly on palpation to discern the fistula(s) features. Furthermore, the larger surface area allowed for the study of haptic exploratory behavior in greater detail. After the subjects palpated, they inserted the needle into the simulated vascular access, during which we assessed the cannulation outcome. Since the outcome of the cannulation procedure, i.e., whether or not stable blood flashback was obtained, is of critical importance, we examined the relationship between palpation quality and cannulation outcomes.

For efficient and effective palpation in clinical settings, clinicians must palpate accurately, i.e., identify points of interest with precision, while taking minimal time. Motivated by this, we examined differences in palpation time between HPs and LPs. Consequently, we hypothesized that our time metrics would be an indicator of palpation skill. In our study, HPs demonstrated statistically significantly shorter palpation *Dwell Time* and *Idle Time* campared to LPs. This result is in contrast to several studies conducted by Pugh and colleagues, who conducted several studies to examine palpation behavior on multiple simulators using sensors. They reported that, for both CBE and pelvic examination via palpation, time taken for palpation did not differentiate HPs from LPs [5], [8], [27]. One exception is a study examining DRE, which indicated that the most experienced group had statistically significantly shorter palpation times than the intermediate group and longer time than the least experienced group [32]. The result of this particular study indicates that there is likely an optimal range of time for a thorough examination. Each of these studies had the goal of palpation for nodule detection. In contrast, Since our study was focused on vascular palpation, times may play a more significant role in cannulation than other examinations.

The location-based metrics for the groups in this paper were previously introduced in Liu *et al*. [36]. However, that study did not attempt to differentiate between HP, MP, and LP users. In this study, *Path Length* was statistically significantly shorter for HP clinicians, likely because HPs can gauge the location of vibration with greater precision and move deliberately towards that area. In contrast, for other types of physical examinations, palpating a larger area is crucial for detection of tissue pathologies; therefore, in some previous studies it would be expected that HPs palpated more area and thus have higher *Path Length* or number of sensors palpated [5], [27], [32]. Our study, for the first time, highlights that expertise in vascular palpation might be related to intentional palpation, which may mean lesser palpation area.

Similarly, HPs had a higher *RCM* than either LP or MP group, suggesting greater intentionality in movement that utilizes knowledge gained from the current touchpoint during palpation to inform the following movement. Since the *RCM* metric measures the percentage of user movements toward the point of “thrill” (location of anastomosis), this metric may be particularly beneficial for real-time training.

The ratio of near touchpoints, *RNTP*, provides insight into the haptic perceptual ability of our users. HPs had statistically significantly fewer overall *Touchpoints* in our study but a higher ratio of touchpoints closer to anastomosis (*RNTP*) than LPs. Alternatively, HPs palpated statistically significantly less than LPs beyond the point of anastomosis (lower *TPD*). Accurate palpation must include discerning blood-flow-based stimuli well since misperception can result in an inaccurate diagnosis or having the needle inserted at an undesirable location. Pugh and Youngblood also reported that high performers touched areas of interest a significantly higher number of times than low performers [27].

This study also extended our previous work by incorporating a force sensor to measure finger forces applied by participants on the simulator. Our results demonstrated that HPs applied statistically significantly lower *Total Force* than either MPs or LPs. In other palpation tasks, force magnitudes were reported as effective in distinguishing between LP and HP palpation skill [8], [27], [32], [42]. For example, Pugh and colleagues’ studies on CBE and DRE reported that HPs apply higher forces than low performers. One exception to this trend is a pilot study by Granados *et al*., which reported no correlation between force and correct diagnosis [30]. However, since the vascular palpation task is qualitatively different from stiffness-based palpation, applying a large magnitude of force is not critical for skilled palpation. Per our results, LPs tend to use larger forces (*TPF*) during palpation, likely because their behavior is more exploratory as they may be unfamiliar with discerning and interpreting vibration resulting from blood flow. HPs, on the other hand, because of their familiarity with vasculature and their ability to rely on vibration stimuli, do not seem to need to apply as much force.

In addition to *TPF*, we also computed the number of *Touchpoints*, *TPT*, *Frequency*, and *Force Range* as other force-based metrics. We seek to quantify palpation behavior further using these force metrics. In our study, HPs had fewer *Touchpoints* than LPs per trial, indicating that they identified fistulas more efficiently. In addition, dwell times (*TPT*) were statistically significantly higher for HPs, indicating that HPs were more intentional with each of their touches. Finally, touchpoint frequency, *TPF* was found to be higher for LPs than HPs. Together, these metrics demonstrate that force measurements can meaningfully quantify and differentiate palpation skill between HPs and LPs. It is also interesting to note that the number of *Touchpoints* and *TPT* are similar to metrics used in an eye-tracking study measuring gaze behavior during laparoscopic surgery [43]. Our results are akin to this study where HPs had fewer “fixations” while holding each “fixation” longer than other performers. Expanding these ideas to palpation, a shorter palpation time with a higher dwell time demonstrates deliberate movement for force perception.

In summary, using the suite of metrics presented in this work, skill differences between the HP, MP, and LP groups are evident. Notwithstanding this, some caveats for the results presented are in order. The differentiation of subjects into high, mid, and low performers limits describing skill into three groups. Skill, however, can be conceptualized as being on a continuum rather than in discrete levels. Further, GRS scores are coarse, with subjects receiving only one score in each category based on sixteen trials. As such, while using GRS scores is commonplace in simulation literature, they only provide a subjective and summative observation of performance.

The following discussion pertains to the relationship between palpation metrics and cannulation outcome measured by the previously published *stb* metric. Because so few of the trials were unsuccessful within the HP group, quantifying the relationship between metrics and outcome, i.e., probability of success, is not tractable. HPs have learned what constitutes skilled palpation, though there may be some stylistic differences in behavior within the group, as seen in variability of the palpation metrics and they ought to continue doing what they have learned. In other words, there is minimal to no room for improvement for their palpation metrics when it comes to stable flashback.

However, successfully obtaining blood flashback is not the only clinically relevant outcome. In an era where patient-centered clinical outcomes are rightly emphasized, the dialysis community is aware of the need to reduce patient pain and anxiety during cannulation. An important facet of patient experience during cannulation is the clinician’s ability to competently assess the vascular access to cannulate efficiently [44], [45]. As a recognition of this, the KDOQI clinical guidelines prescribe that all cannulators perform a “look-listen-feel” test, of which palpation (feel) is an indispensable component [13]. While the aforementioned result suggests that HPs palpation behavior does not affect the probability of cannulation success, there can certainly be room for improvement in their palpation technique. For instance, if an HP’s palpation forces are higher than most participants, this individual could benefit from learning to palpate more gently. This has important implications for patient comfort, pain, and anxiety [44], [45]. Furthermore, this approach moves training to truly be patient-centered by considering patient experience as well as clinical outcomes.

One of the most salient benefits of a simulator is its potential to train the skill of novice or unskilled trainees. In our study, the LP group approximates novice trainees. As such, we are interested in identifying specific palpation metrics that statistically significantly predict cannulation success. As can be noted from Table 2 four palpation metrics: *Idle Time*, average *TPT*, *Touchpoints*, and *Frequency* statistically significantly predicted cannulation success. We suggest that these four metrics could be a potential starting point for designing training strategies. For instance, average *TPT* significantly predicts *p*(*success*). Specifically, if the average *TPT* is greater than one second, the likelihood of success is markedly higher, reaching *p*(*success*) > 0.8. That is, trainees learning cannulation can be encouraged to spend more time feeling the fistulas with quantitative feedback available through the simulator. Other metrics that significantly predict cannulation success (*Idle Time*, *Total Force*, and *Frequency*) could also be used in formulating directed feedback for palpation skill training.

MPs also have metrics that become statistically significant for predicting success. It is worth noting that MPs improved with increased frequency, whereas LPs got worse with increased frequency. It could be argued that this is the result of the inherent skill of MPs that allows them to accomplish the task well at high frequency as opposed to LPs who should be slowing down, walk before you run, as it were. For MPs, two metrics not seen in the LP models become statistically significant, implying that other metrics are related to cannulation success for people with the MP skill level. In summary, these results provide critical insights into the relationship between process metrics and cannulation outcomes that could be useful for skill assessment and training.

## V. CONCLUSION

Palpation is an essential step for clinical examinations and procedures. In this study, the goal was to ascertain using a cannulation simulator for palpation assessment using objective metrics to differentiate HPs and LPs. This work provides validity evidence for the simulator’s capability to differentiate palpation skill among high and low performing cannulators objectively. HPs completed the task more confidently and intentionally, taking shorter amounts of time, having a higher ratio of correct movement, and having a shorter *Path Length*. These metrics can be applied in the training of palpation skill by providing objective quantification of palpation behavior. In the future, we intend to further study the simulator’s validity for training both on the simulator and in the clinical environment, including a comprehensive score for vascular palpation, following clinical guidelines [46].

## Figures and Tables

**FIGURE 7.**
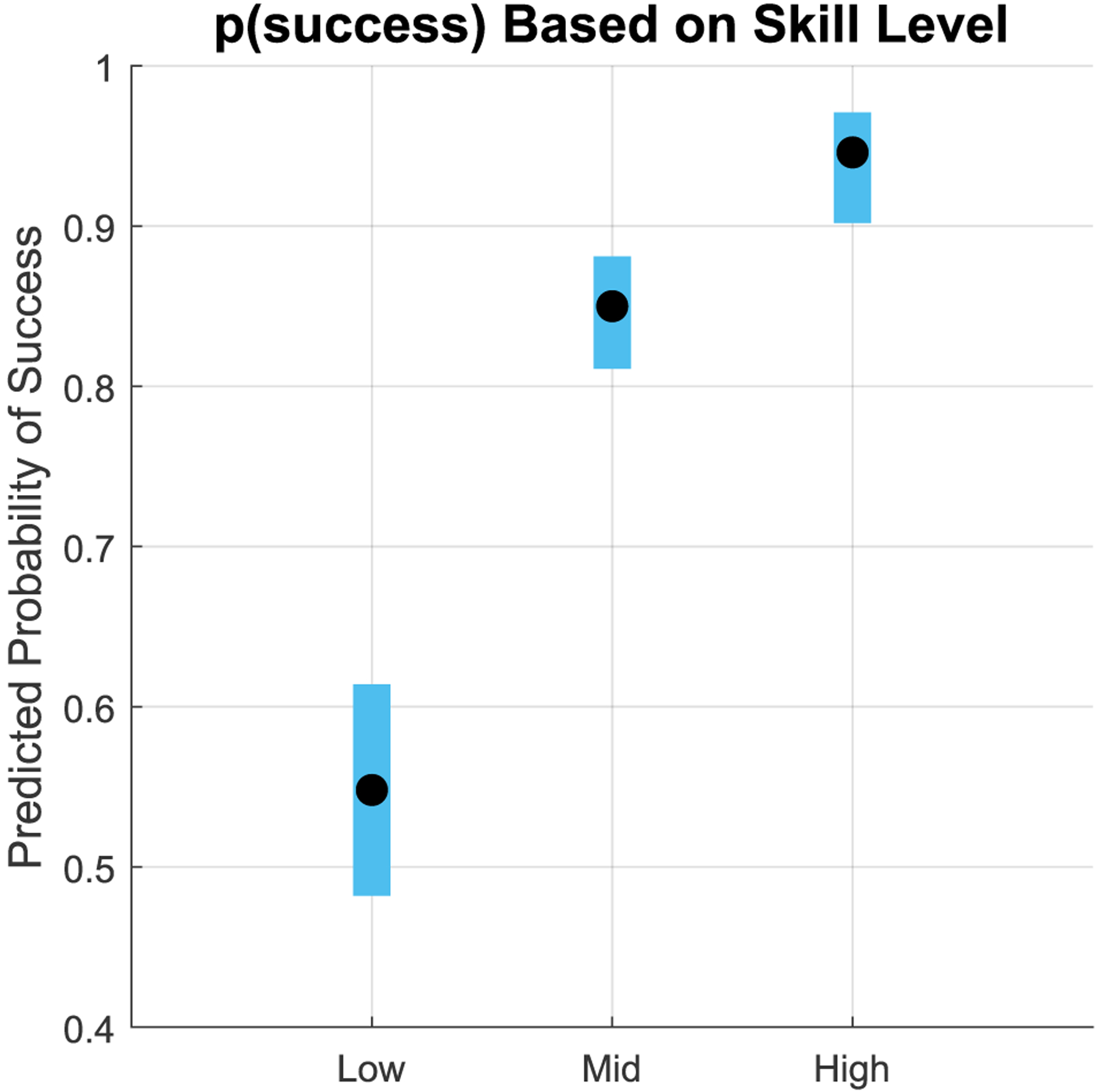
**Plot of the predicted probability of success based on skill level**.

**TABLE 1.** Summary of statistical results showing the means of the groups and the result of Mann-Whitney tests comparing HPs, MPs, and LPs.

	Metric	LP v HP	LP v MP	MP v HP
Time	*Idle Time* (s)	**6.73 v 5.05** ***	6.73 v 6.04	**6.04 v 5.05** *
	*Dwell Time* (s)	**8.96 v 5.68** ***	**8.96 v 6.70** **	**6.70 v 5.68** **
Location	*Path Length* (mm)	**1373 v 871** ***	**1373 v 1128** *	**1128 v 871** *
	*RCM* (%)	**66.7 v 75** *	66.7 v 66.7	**66.7 v 75** *
	*RNTP* (%)	**12.1 v 27.9** *	**12.1 v 22.2** *	22.2 v 27.9
	*TPD* (mm)	**81.0 v 54.0** ***	81.0 v 80.9	**80.9 v 54.0** ***
Force	*Touchpoints*	**20 v 13** ***	**20 v 15** **	**15 v 13** *
	*Total Force* (N)	**8.94 v 3.71** ***	**8.94 v 6.25** **	**6.25 v 3.71** ***
	*Force Range* (N)	**1.28 v 1.00** ***	1.28 v 1.23	**1.23 v 1.00** ***
	*Frequency* (Hz)	1.21 v 1.19	1.21 v 1.16	1.16 v 1.19
	*TPT* (s)	**0.326 v 0.350** ***	**0.326 v 0.386** **	**0.386 v 0.350** *
	*TPF* (N)	**0.305 v 0.267** ***	**0.305 v 0.322** **	**0.322 v 0.267** **

* p-value <0.05

** p-value <0.01

*** p-value <0.001

**TABLE 2.** Coefficients of the stratified uni-variate logistic regression Force models.

Metric	LP	MP	HP
*Idle Time*	**−0.09415** ***	−0.03659	0.08877
*Dwell Time*	−0.1782	**−0.04205** **	0.01215
*Avg TPT*	**2.6702** ***	−0.7322	−0.83
*Touchpoints*	**−0.05262** ***	−0.0137	0.0308
*Total Force*	0.004	**−0.01877** *	0.00358
*Force Range*	−0.2230	0.1214	−0.00985
*Frequency*	**−1.2012** ***	**0.7927** *	1.27

* p-value <0.05

** p-value <0.01

*** p-value <0.001
